# Metabolic network modeling of redox balancing and biohydrogen production in purple nonsulfur bacteria

**DOI:** 10.1186/1752-0509-5-150

**Published:** 2011-09-25

**Authors:** Oliver Hädicke, Hartmut Grammel, Steffen Klamt

**Affiliations:** 1 Max Planck Institute for Dynamics of Complex Technical Systems, Sandtorstrasse 1, D-39106 Magdeburg, Germany; 2 MaCS - Magdeburg Centre for Systems Biology, Sandtorstrasse 1, D-39106 Magdeburg, Germany

## Abstract

**Background:**

Purple nonsulfur bacteria (PNSB) are facultative photosynthetic bacteria and exhibit an extremely versatile metabolism. A central focus of research on PNSB dealt with the elucidation of mechanisms by which they manage to balance cellular redox under diverse conditions, in particular under photoheterotrophic growth.

**Results:**

Given the complexity of the central metabolism of PNSB, metabolic modeling becomes crucial for an integrated analysis of the accumulated biological knowledge. We reconstructed a stoichiometric model capturing the central metabolism of three important representatives of PNSB (*Rhodospirillum rubrum, Rhodobacter sphaeroides *and *Rhodopseudomonas palustris)*. Using flux variability analysis, the model reveals key metabolic constraints related to redox homeostasis in these bacteria. With the help of the model we can (i) give quantitative explanations for non-intuitive, partially species-specific phenomena of photoheterotrophic growth of PNSB, (ii) reproduce various quantitative experimental data, and (iii) formulate several new hypotheses. For example, model analysis of photoheterotrophic growth reveals that - despite a large number of utilizable catabolic pathways - substrate-specific biomass and CO_2 _yields are fixed constraints, irrespective of the assumption of optimal growth. Furthermore, our model explains quantitatively why a CO_2 _fixing pathway such as the Calvin cycle is required by PNSB for many substrates (even if CO_2 _is released). We also analyze the role of other pathways potentially involved in redox metabolism and how they affect quantitatively the required capacity of the Calvin cycle. Our model also enables us to discriminate between different acetate assimilation pathways that were proposed recently for *R. sphaeroides *and *R. rubrum*, both lacking the isocitrate lyase. Finally, we demonstrate the value of the metabolic model also for potential biotechnological applications: we examine the theoretical capabilities of PNSB for photoheterotrophic hydrogen production and identify suitable genetic interventions to increase the hydrogen yield.

**Conclusions:**

Taken together, the metabolic model (i) explains various redox-related phenomena of the versatile metabolism of PNSB, (ii) delivers new hypotheses on the operation and relevance of several metabolic pathways, and (iii) holds significant potential as a tool for rational metabolic engineering of PNSB in biotechnological applications.

## Background

Purple nonsulfur bacteria (PNSB; Rhodospirillaceae) are widely used as model organisms in microbiology and, to an increasing extent, for systems biology. They were extensively studied with respect to the molecular structure of their photosynthetic apparatus [1] and draw further attention of research activity due to their outstanding metabolic versatility and adaptability [2-8]. Aerobically in the dark, they grow chemoheterotrophically by respiration. When oxygen becomes limiting and light is available, these facultative photosynthetic bacteria respond by synthesizing an extensive system of light-capturing intracytoplasmic membranes. They then switch to photoheterotrophic growth (with an organic substrate as carbon and electron source) or to photoautotrophic growth (if CO_2 _and an inorganic electron donor such as hydrogen are supplied). PNSB possess the well-known Calvin-Benson-Bassham cycle (Calvin cycle) which is essential for autotrophic but partially also for photoheterotrophic growth [5,6,8-11]. In darkness without oxygen they can grow by fermentation or anaerobic respiration. The ability to utilize di-nitrogen (N_2_) as source of organic nitrogen (via a nitrogenase) further demonstrates the wide spectrum of metabolic abilities of these bacteria [12-14]. Given this extraordinary metabolic versatility, the elucidation of key mechanisms enabling these organisms to switch between different lifestyles thereby maintaining redox balance has been an important focus of research [5,10,11,15-18].

Stoichiometric network analysis based on the constraint-based modeling framework has been proven to be a valuable tool to study cellular metabolism and phenotypic capabilities of many organisms [19]. Given the complexity of the central metabolism of PNSB, metabolic modeling becomes a key for an integrated analysis of the accumulated biological knowledge and it may help to gain a deeper and holistic understanding of the metabolism in PNSB. There is a large set of analytical methods available for constraint-based modeling including metabolic flux analysis (MFA) [20,21], flux balance analysis (FBA) [22-24] and metabolic pathway analysis (e.g. based on elementary modes [25]). All these methods rely solely on the stoichiometric structure and thermodynamic (i.e. reversibility) constraints of reactions and they do not require knowledge of kinetic mechanisms and parameters. To reconstruct organism-specific metabolic networks, available information on annotated genomes and established biochemical knowledge can be used.

In this study we will present an extended and manually curated stoichiometric network model of the central metabolism of PNSB and use it to explain a number of observed phenomena related to PNSB metabolism. While reconstructing this network, we concentrated on three organisms where the amount of available data is sufficient to set-up a stoichiometric model of the core metabolism. The chosen representatives are *Rhodospirillum (Rs.) rubrum*, *Rhodobacter (Rba.) sphaeroides *and *Rhodopseudomonas (Rps.) palustris*. Although we concentrate on these representatives, the model is of general relevance for the whole family of PNSB. At the same time, the integration of three models into a single stoichiometric "master" model allows comparative studies of interesting differences in central metabolic pathways. For example, the enzymatic composition of the Calvin cycle and of the glycolytic (Embden-Meyerhof-Parnas) pathway are identical in all three organisms but there are differences in alternative hexose breakdown pathways such as the oxidative branch of the pentose phosphate pathway (only present in *Rps. palustris*) or the Entner-Doudoroff pathway (only present in *Rba. sphaeroides*; see Methods section). Furthermore, only *Rs. rubrum *has been shown to possess two enzymes (pyruvate synthase and α-ketoglutarate synthase) of the "reductive carboxylic acid cycle" using reduced ferredoxin as reductant [26,27].

Using flux variability analysis, we will analyze the reconstructed model with respect to general (PNSB-wide) and species-specific network properties and utilize it to characterize phenotypic states for different environmental conditions. Particular attention will be paid to aspects of redox balancing and redox metabolism during photoheterotrophic growth. Besides, we will also examine peculiarities of acetate assimilation in these organisms which has recently attracted considerable interest [28-33] since *Rs. rubrum *and *Rba. sphaeroides *lack isocitrate lyase (in contrast to *Rps. palustris*), and can thus not utilize the well-known glyoxylate shunt. An alternative assimilation route discovered recently is the ethylmalonyl-CoA pathway [29]. Another proposed pathway is the citramalate cycle [31]. We will use our model to find out which of the two putative pathways can best explain experimental observations made under photoheterotrophic conditions.

## Results and Discussion

### A stoichiometric model of the central metabolism of PNSB

**Figure 1:**
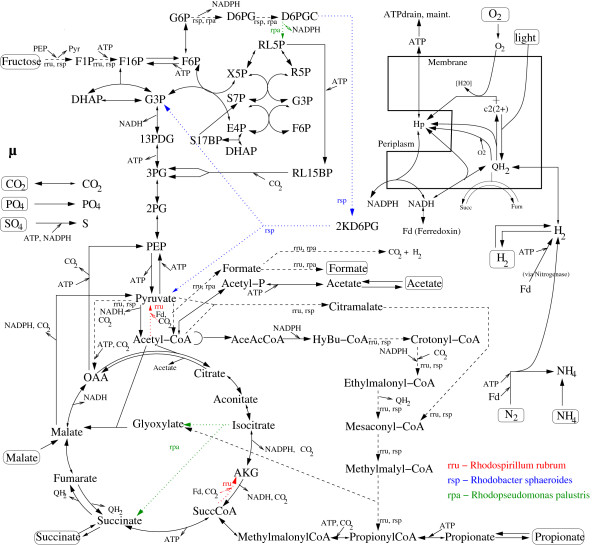
**Central metabolism of purple non-sulfur bacteria (catabolic and amphibolic reactions)**. Reactions that are specific for *Rs. rubrum *(rru; red dotted lines), *Rba. sphaeroides*, (rsp, blue dotted lines) and *Rps. palustris *(rpa; green dotted line) are indicated. Reactions with dashed lines occur only in two of the three representatives (indicated by their abbreviations). Solid lines indicate reactions present in all three species.

We manually reconstructed a metabolic "master" model of the major catabolic pathways of the PNSB representatives *Rs. rubrum*, *Rba. sphaeroides *and *Rps. palustris *(Figure 1; see Methods section for details). For several reasons we intentionally refrained from constructing a genome-scale model and aimed instead at building a detailed and manually curated model of the core metabolism of PNSB. First, the focus of this work is clearly on aspects of the central carbon metabolism and a genome-scale model would therefore be out of the scope of our intended analysis (even more when being interested in a "master" model of three different species to allow for comparative studies). Our approach is thus similar to ^13^C labeling studies [34] characterizing different metabolic states by exploring the role and possibly (re-)distribution of major metabolic fluxes in the central metabolism under different environmental conditions. Furthermore, a fully automatic generation of a genome-scale model using tools such as ModelSEED [35] will not be possible since several "exotic" metabolic pathways such as the recently discovered ethylmalonyl-CoA pathway are not contained yet in databases such as KEGG.

We included catabolic and amphibolic pathways (including substrate uptake and product excretion) as complete as possible by carefully screening available biochemical literature and databases. In contrast, biosynthetic routes for the production of building blocks and macromolecules were modeled in a simplified manner by using lumped reactions. This representation is still fully sufficient to compute quantitatively precursor effluxes into biomass synthesis and to include a biomass synthesis reaction accordingly. We do not expect that the key results of network analysis presented herein would change qualitatively when taking a genome-scale model instead.

Since the network model was reconstructed as a master model, there exist some reactions that were only available in one or two of the three species. These were marked accordingly in Figure 1 (each species possess two reactions not contained in the other two representatives). The reconstructed master model consists of 143 reactions and 119 balanced metabolites. For specific details of the metabolic network model we refer to the Methods section. The complete network definition together with the biomass composition can be found in the Additional File 1. The model is also provided in SBML format in the Additional File 2.

### Model analysis

Using the stoichiometric model in combination with flux variability analysis (FVA; see Methods), we will discuss key characteristics of feasible metabolic flux distributions in PNSB for different environmental *scenarios*. Each scenario is characterized by the chosen substrate and a set of reaction rates fixed to zero reflecting the environmental conditions (Table 1). For example oxygen uptake is zero under (anaerobic) phototrophic conditions. Specific constraints will be explained when discussing the scenarios. Generally, we normalize the uptake rate of the chosen substrate to unity whereas all other substrate uptake rates are set to zero (the validity of this normalization approach is explained when discussing the scenarios). As no significant amounts of excreted products (besides CO_2_) are produced under phototrophic as well as aerobic conditions [6] the excretion of products except CO_2 _is disabled (rate set to zero) by default but will be allowed when simulating fermentative metabolism. Nitrogenase and hydrogen release were considered as inactive if not stated otherwise. As explained in the Methods section, we then employ flux variability analysis to investigate the resulting space of feasible flux distributions. Particular attention will be paid to reaction rates that are uniquely determined (i.e. if upper and lower boundaries as computed by FVA coincide).

**Table 1:** Flux constraints set for the different environmental scenarios discussed in the main text.

Pathway/Reaction	S1 general photoheterotrophic	S2 constrained photoheterotrophic	S3 photohet. on acetate with glyoxylate cycle	S4 photohet. on acetate with CM cycle	S5 photohet. on acetate with EMCoA pathway	S6 anaerobic in the dark	S7 aerobic in the dark
N2_up	0	0	0	0	0	0	0
O2_up	0	0	0	0	0	0	
Succ_ex	0	0	0	0	0		0
Prop_ex	0	0	0	0	0		0
Ac_ex	0	0	0	0	0		0
Form_ex	0	0	0	0	0		0
Hydrogen release (H2_ex)	0	0	0	0	0	0	0
Photosynthesis (Photo)						0	0
Calvin cycle (Rubisco)							0
oxidative pentose-phosphate pathway (PGluc::Rl5P)		0					
Entner-Doudoroff (PGluc::KDPG)		0					
oxidative TCA (AlKG::SuccCoA)		0	0	0	0		
reductive TCA (SuccCoA::alKG; AcCoA::Pyr)		0	0	0	0		0
Fumarate reductase (Fum::Succ)							0
Glyoxylate cycle (ICit::SuccGlyox)		0		0	0		
CM cycle (CitMal_synth)		0	0		0		
EMCoA pathway (HyBuCoA::CrotCoA)		0	0	0			

In addition to the listed constraints, the uptake rate of the chosen substrate is set to 1 and all remaining substrate uptake rates are set to zero (not listed in the table). Considered substrates are succinate, malate, fructose (for S1, S2, S6 and S7), propionate (for S1, S2 and S7), and acetate (for S1, S3, S4, S5 and S7). Autotrophic growth (on CO_2 _and hydrogen mixture) was also considered for S1 and S2.

With the chosen scenarios we will discuss several issues related to cellular redox balance under photoheterotrophic conditions in PNSB. We will also investigate the role of the proposed alternative acetate assimilation pathways. To highlight the differences to photoheterotrophic growth conditions, some theoretical aspects of aerobic and fermentative growth in the dark will be briefly discussed afterwards.

### Photoheterotrophic growth on different substrates: redox balancing poses tight constraints on carbon metabolism (scenario S1)

Three observed key characteristics of photoheterotrophic growth of PNSB are (i) the CO_2 _production rate correlates with the oxidation state of the chosen substrate; (ii) usually no other products than CO_2 _can be detected under optimal growth conditions [6] and (iii) some of the released CO_2 _is reused via CO_2 _fixation pathways. Using our metabolic model we first try to understand observation (i) taking observation (ii) into account. We start with succinate as carbon source. Scenario S1 (Table 1) defines the most general case for photoheterotrophic growth on this substrate with an uptake rate normalized to 1 mmol/(gDW⋅h). Rates fixed to zero simply reflect that neither oxygen nor other substrates are available and that no products are excreted. Mathematically, this scenario is extremely underdetermined as the number of unknown reaction rates is much larger than the number of equations posed by eq. (1) (31 degrees of freedom). The *in silico *cell can potentially utilize a huge number of possible combinations of the available pathways: Calvin cycle, reductive or oxidative TCA, Entner-Doudoroff, glycolysis, (oxidative and non-oxidative) pentose phosphate pathways, several anaplerotic reactions and even the three pathways for acetate assimilation (glyoxylate shunt, citramalate pathway, ethylmalonyl-CoA pathway) are enabled. Furthermore, as explained in the Methods section, we do not assume optimal growth or other biological objective functions as typically used in FBA studies. However, applying FVA, the unexpected result is that the growth rate *μ *and thus the biomass yield as well as the CO_2 _excretion rate are uniquely determined (Table 2). Since we considered the master network containing the reactions and pathways available in *Rs. rubrum *or/and in *Rba. sphaeroides*, or/and in *Rps. palustris*, this result holds for all three species and can be seen as a general result for PNSB: further constraining scenario S1 by setting some additional reactions to zero (e.g., because they are not available in one of the representatives) cannot affect this result as such a scenario would only be a special (more constrained) case of S1. Note that we normalized the substrate uptake rate to unity. The real uptake rate will depend on a number of factors we cannot consider in a stoichiometric model (e.g. light intensity, substrate concentration). However, in order to get the absolute values of the growth and CO_2 _excretion rate, we can here simply multiply the computed normalized values with the (measured) real uptake rate. Importantly, the computed biomass (gDW per mmol substrate) and CO_2 _(mmol per mmol substrate) yields given in Table 1 are invariant to the uptake rate (note that this holds only because redox and energy (including maintenance) metabolism are stoichiometrically decoupled; see below). Furthermore, we observed that a moderate change of the biomass composition affects the computed values only slightly (data not shown). In any case, changing the biomass composition cannot abolish the fact that there are unique rate ratios for each substrate.

**Table 2:** Model-based predictions and experimental data on net CO_2 _release, biomass yield and RubisCO flux for photoheterotrophic growth of PNSB on different substrates.

	Succinate	Malate	Acetate	Fructose	Propionate	Hydrogen (autotroph)
						

***Net CO2 release***	*Scenario S1/S2*	*Scenario S1/S2*	*Scenario S1/S3/S4/S5*	*Scenario S1/S2*	*Scenario S1/S2*	*Scenario S1/S2*

calculated [mmol CO_2_/mmol substrate]	0.72	1.19	0.13	0.38	-0.28	-0.47
([mmol CO_2_/C in substrate])	(0.181)	(0.300)	(0.064)	(0.064)	(-0.094)	(∞)
measured [mmol CO_2_/mmol substrate]	0.75 [37]	1.15 [37]	0.11 [10]		-0.23 [37]	
	0.70 [36]	1.22 [36]	0.25 [37]			
			0.17 [36]			

						

***Biomass yield Y***_***X/S***_	*Scenario S1/S2*	*Scenario S1/S2*	*Scenario S1/S3/S4/S5*	*Scenario S1/S2*	*Scenario S1/S2*	*Scenario S1/S2*

calculated [gDCW/mmol substrate]	0.072	0.062	0.041	0.124	0.072	0.010
calculated [gDCW/g substrate]	0.610	0.463	0.683	0.688	0.973	5.000
measured [gDCW/g substrate]		0.476 [52]	0.660 [44]			
			0.650[10]			

						

***RubisCO flux***	*Scenario S2*	*Scenario S2*	*Scenario S3/S4/S5*	*Scenario S2*	*Scenario S2*	*Scenario S2*

Calculated minimal *RubisCO *flux [mmol/mmol substrate]	0.73	0.20	S3: 0.22	0.40	0.73	0.53
			S4: 0.22			
			S5: 0			
[mmol/mmol C in substrate]	(0.184)	(0.05)	S3: 0.11	(0.067)	(0.245)	(∞)
			S4: 0.11			
			S5: 0			
Measured [mmol/mmol substrate]			S3: 0.29[10]			

The data taken from references [36] and [37] are averaged values over replicated experiments.

Why does a given substrate uptake rate imply a fixed biomass and CO_2 _yield irrespective of the pathways used (if no products except CO_2 _are released)? The answer is strongly linked to cellular redox balance: given a substrate uptake rate, only the computed biomass and CO_2 _yields will enable the cell to balance reducing equivalents (NAD(P)H) and carbon metabolites simultaneously thereby changing the oxidation level of the substrate carbon to the oxidation level of the biomass. This result also implies that all available alternative pathways (and cycles) are equivalent with respect to the consumed/produced carbon and reducing equivalents (otherwise the yields could not be determined uniquely). This tight coupling between redox and carbon balance is a key property of phototrophic growth of PNSB: under these conditions, energy production in form of ATP is decoupled from redox cofactors (NAD(P)H) since cyclic photosynthesis does neither consume nor produce electrons - in contrast to respiration where oxygen serves as a flexible redox sink (see below). In other words, we would obtain the same results for biomass and CO_2 _yields even when not balancing ATP in our model.

We repeated the scenario S1 for other substrates revealing the same fundamental constraints (Table 2): biomass and CO_2 _yield are fixed, though specific values result for each substrate. The net CO_2 _release clearly correlates with the oxidation state of the respective substrate confirming general observations [6,8,10,36]. The highest net CO_2 _release rate can be found for the most oxidized substrate malate, while the reduced substrate propionate implies a negative net production (i.e., a net fixation of CO_2_) confirming the observation that PNSB can only grow on propionate if bicarbonate is provided with the medium [37,38]. The computed biomass and CO_2 _yield agree remarkably well with measurements found in the literature (Table 2) confirming this inherent network property. Obviously, would we allow the excretion of certain products (e.g. formate or acetate), biomass and CO_2 _yields would not be fixed anymore; however, the good agreement of our predictions with measurements in Table 2 confirms that product excretion is usually not observed under photoheterotrophic conditions (at least under high-light conditions).

Note that similar findings as discussed in this section were also mentioned by us in [20]. However, the model presented herein is significantly larger (143 vs. 76 reactions) and therefore generalizes the results shown in [20].

### Photoautotrophic growth

Under photolithoautotrophic conditions with a CO_2_/H_2 _mixture, hydrogen serves as electron donator and CO_2 _as carbon source and electron acceptor. With biomass as the only product we adapt scenario S1 to represent autotrophic growth: this time we normalize the H_2 _uptake rate to 1 mmol/(gDW⋅h). Again, as for the photoheterotrophic case, we can uniquely determine the resulting biomass yield and the CO_2 _net consumption (per mmol H_2 _taken up; Table 2). It is clear that under these conditions CO_2 _must be fixed and the ratio of CO_2 _fixed per H_2 _is, again, governed by the oxidation state of the biomass.

### The role of the Calvin cycle under photoheterotrophic conditions (scenario S2)

The physiological role of the Calvin cycle for photoheterotrophic growth has been a central subject of biochemical research in PNSB [5,8-11,14,18,39,40]. This interest was driven by the unintuitive observation that PNSB require the Calvin cycle (or exogenous electron acceptors) even for photoheterotrophic conditions on substrates where a net CO_2 _release occurs (see measurements given in Table 2). It was hypothesized that the Calvin cycle under photoheterotrophic conditions might primarily serve as a sink for redox equivalents. The net stoichiometry of the Calvin cycle reads: 3 CO_2 _+ 6 NADH + 9 ATP -> 3PG + 6 NAD + 9 ADP + 8 Pi (note that the Calvin cycle in PNSB consumes NADH in contrast to plants where NADPH is consumed). Thus, for each 3-phosphoglycerate (3PG) synthesized, 6 NADH are oxidized by the Glyceraldehyde-3-phosphate (G3P) dehydrogenase reaction acting in direction of G3P.

Another unexpected behavior is for example, that *Rba. sphaeroides *mutant strains lacking ribulose-1,5,-bisohosphate carboxylase/oxygenase (RubisCO), the key enzyme of the Calvin cycle, could not grow photoheterotrophically on malate [9,33,39], whereas a RubisCO mutant of *Rs. rubrum *could [5,9,27,40].

**Figure 2:**
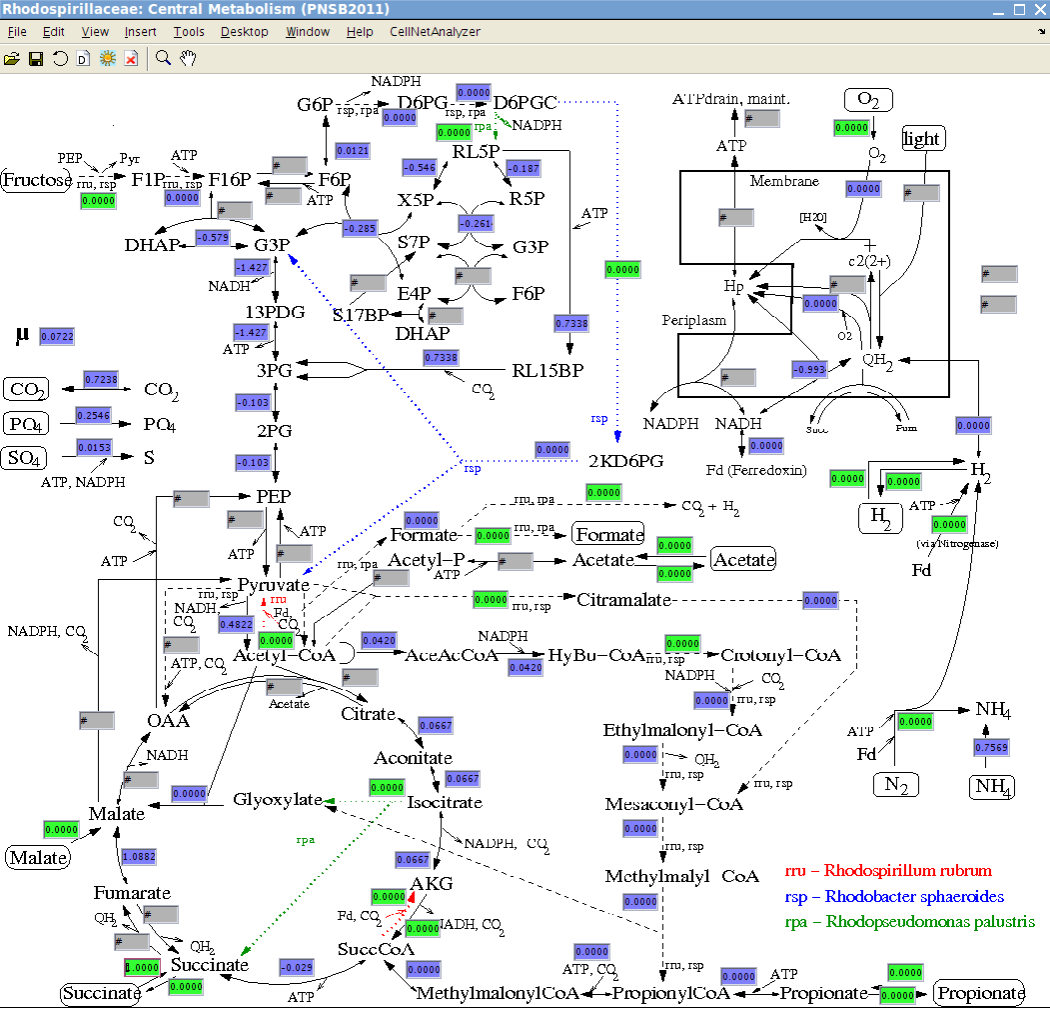
**Resolvable reaction rates resulting for scenario S2 (screenshot of *CellNetAnalyzer*)**. The depicted scenario S2 simulates photoheterotrophic growth on succinate normalized for a substrate uptake of unity. Green boxes: prescribed constraints of S2 (cf. Table 1); blue boxes: uniquely calculable reaction rates (upper and lower bound of a reaction rate coincides in FVA); gray boxes: undetermined fluxes.

In the scenario S2 discussed in the following we take scenario S1 and fix initially some additional reaction rates to zero reflecting reasonable assumptions for growth on succinate (Table 1; Figure 2):

(i) We disable the oxidative pentose phosphate pathway (from *Rps. palustris*) and Entner-Doudoroff pathway (from *Rba. sphaeroides*). This corresponds directly to the situation in *Rs. rubrum *which cannot use any of these two routes. These pathways are also unlikely to operate in *Rb. sphaeroides *and *Rps. palustris*, respectively, during growth on substrates that require gluconeogenesis (see e.g. [10], where a very low flux through the glucose-6-phosphate dehydrogenase reaction was determined).

(ii) The two ferredoxin-dependent key enzymes of the reductive TCA are not available in *Rba. sphaeroides *and *Rps. palustris*. They have been shown to exist in *Rs. rubrum *but seem to exhibit relatively low activity under photoheterotrophic conditions [27]. We therefore assume that the low flux through the α-ketoglutarate synthase neutralizes the reverse flux through the of α-ketoglutarate dehydrogenase (exhibiting a low activity under photoheterotrophic conditions; see e.g. [41]) and set both fluxes to zero.

iii) We consider the glyoxylate shunt and the two putative alternative acetate assimilation pathways (citramalate cycle and ethylmalonyl-CoA pathway) to be inactive when growing on substrates different from acetate.

The potential role of the oxidative pentose phosphate pathway, the Entner-Doudoroff pathway and of the reductive TCA will be discussed in a subsequent section. Likewise, the role of the acetate assimilation pathways will also be studied separately.

We then apply again FVA to identify reaction rates that can be uniquely deduced for this scenario S2. As expected, for the rates calculable in scenario S1 (Table 2; Figure 2) we get the same values also in S2 because scenario S2 is a special case of S1. Furthermore, we find that some rates undetermined in S1 can now also be assigned a fixed value. To be more specific, 95 of the 143 fluxes are now uniquely calculable, many of them corresponding to anabolic fluxes resulting from the growth rate. 18 rates are trivially calculable as zero fluxes (they are in a linear pathway with a reaction that was fixed to zero by the applied constraints). The remaining calculable fluxes lie within their feasible intervals (and none of them is at its min/max boundary). One of the newly calculable reaction rates is the flux of RubisCO which, despite of a net release of CO_2_, is relatively high: about 50% of the *total *release of CO_2 _(produced e.g. by anaplerotic reactions such as PEP carboxykinase or malic enzyme) reenters the metabolism via the Calvin cycle and the RubisCO flux is therefore approximately as high as the *net *release of CO_2_. Our network analysis thus confirms the hypothesis that, under photoheterotrophic conditions, the Calvin cycle is primarily required for maintaining redox balance (rather than for providing additional carbon - which of course is its main function during photoautotrophic growth): it serves as a sink for electrons being produced in excess when metabolizing succinate, notably due to the activity of succinate dehydrogenase (in combination with reverse electron flow to NADH dehydrogenase) and malate dehydrogenase. Although a substantial amount of reducing equivalents is required for biomass synthesis, the amount of electrons generated by succinate utilization exceeds this biosynthetic demand. Not all rates of the reactions in the Calvin cycle could be determined uniquely in S2, however, Figure 2 shows that the reaction of the Glyceraldehyde-3-phosphate dehydrogenase consumes considerable amounts of NADH and that there is also a gluconeogenic flux from PEP which (in addition to the net synthesis of 3PG in the Calvin cycle) contributes to the synthesis of precursors in the Embden-Meyerhof and the pentose-phosphate pathway.

Except for acetate (see below), we simulated scenario S2 also for the other substrates (Table 2). The results clearly show that a non-zero flux through the Calvin cycle is required for all organic substrates considered; the total amount of the flux depends on the oxidation values of the substrates *and *on structural constraints imposed by the pathways initially metabolizing the substrate. For example, growth on malate requires less RubisCO flux than growth on succinate because malate is more oxidized than succinate and generates therefore less reducing equivalents when metabolized. On the other hand, although propionate is more reduced than succinate, the same RubisCO flux as for succinate is calculated. This is because propionate is oxidized by the propionyl-CoA carboxylase reaction during the early steps of its utilization leading indeed to a net CO_2 _fixation (Table 2) but requiring no more RubisCO flux as for succinate.

### Photoheterotrophic acetate metabolism (scenarios S3 - S5)

For the case of photoheterotrophic growth with acetate we have to distinguish three different routes for metabolizing acetate. *Rps. palustris *uses the glyoxylate shunt whereas *Rs. rubrum *and *Rba. spharoides *cannot employ this pathway due to the missing isocitrate lyase. An alternative assimilation route discovered recently is the ethylmalonyl-CoA (EMCoA) pathway [29]. Another hypothetical mechanism proposed by [32] and [31] is the citramalate (CM) cycle. We mention here that, in principle, acetate assimilation in *Rs. rubrum *would also be possible via the ferredoxin-dependent pyruvate synthase reaction. However, a simulation shows that, without CM cycle and EMCoA pathway, this would only be possible if the TCA cycle can run in oxidative direction in order to generate the required reducing equivalents (an unlikely event under anaerobic conditions). Therefore, also because *Rba. sphaeroides *does not possess this enzyme, we will set the flux of the pyruvate synthase to zero. As a somewhat surprising result, the model also shows that, in principle, photoheterotrophic acetate assimilation would even be possible without pyruvate synthase and without all three (EMCoA pathway, CM and glyoxylate cycle) anaplerotic reaction sequences. This could be achieved by a joint operation of the oxidative TCA (oxidizing acetyl-CoA, yielding CO_2 _and NADH) and the Calvin cycle (re-reducing CO_2 _with NADH); the latter operating as the anaplerotic source for supplying precursor metabolites upstream of the TCA. However, this operation is energetically wasteful and would again require significant oxidative fluxes in the TCA. Hence, we do not consider this variant as viable.

In order to analyze functional properties of the three proposed acetate assimilation routes, we first summarize their overall stoichiometries. For simplicity, we balance only carbon metabolites and reducing equivalents. In addition, we assume in these net balances that electrons carried to ubiquinone via membrane-bound enzyme complexes such as succinate dehydrogenase will be shuttled to NADH via reverse electron flow (consuming proton motive force (pmf) [42]). Starting with acetyl-CoA and following the pathways up to the synthesis of the intermediate malate, the net stoichiometries read as follows:

i) glyoxylate cycle (*Rps. palustris*):

4 Acetyl-CoA+4 NAD+<pmf>→2 Malate+4 NADH+4 CoA

ii) citramalate-cycle (hypothetical pathway proposed for *Rba. sphaeroides*/*Rs. rubrum*):

4 Acetyl-CoA+4 NAD+<pmf>→2 Malate+4 NADH+4 CoA

(this net balance accounts for the resynthesis of one molecule of pyruvate required for the initial step of the citramalate cycle)

iii) ethylmalonyl-CoA-pathway (proposed for *Rba. sphaeroides*/*Rs. rubrum*):

$$\text{3 Acetyl}-\text{CoA}+\text{2 C}{\text{O}}_{\text{2}}+\text{2 NADPH}+\text{2 NAD }+<\text{pmf}>\to \text{2 Malate}+\text{2 NADH}+\text{2 NADP}+\text{3 CoA}$$

Apparently, the net stoichiometries for the glyoxylate shunt and for the CM cycle are identical. Moreover, whereas the latter two generate two reducing equivalents per molecule malate produced, the EMCoA pathway exhibits a neutral balance regarding the reducing equivalents (one NADH molecule generated per molecule NADPH consumed). Consequently, the EMCoA pathway can still not serve as a redox sink in the overall stoichiometry. Again, this result follows when assuming that electrons carried to ubiquinone in intermediate steps of the pathway will be further conveyed to NADH via reverse electron flow. It is reasonable to take the electrons in the electron transport chain into account in the overall redox balance as they are neither consumed during cyclic photosynthesis nor do we assume that there are other external redox sinks such as oxygen (and release of hydrogen is also excluded at this stage).

As starting point for simulating growth on acetate we used again scenario S2 as in the previous section but re-enabled (separately) the three proposed assimilation pathways (S3: glyoxylate shunt, S4: CM cycle, S5: EMCoA pathway).

Our first observation confirms the results from scenario S1 regarding net biomass synthesis and CO_2 _net release per substrate taken up (Table 2): these values remain fixed irrespective of the assimilation pathway used, hence, measurements thereof cannot be used to discriminate between these pathways. However, there is a striking difference in the minimal required flux through the RubisCO reactions: in case of the glyoxylate shunt and the CM cycle, we compute a minimum RubisCO flux of 0.22 mmol per mmol acetate taken up (Table 2). Thus, analogous to the substrates discussed in the previous sections, the Calvin cycle (or any other redox balancing mechanism; see also the following section) would be needed for a balanced operation of these assimilation cycles. In contrast, the EMCoA pathway implies a minimum RubisCO flux of zero, hence, it can operate without a functional Calvin cycle. As already pointed out by Laguna et al. [33], one reason is that the glyoxylate and CM cycle produce reducing equivalents whereas the EMCoA pathway consumes reducing equivalents (and CO_2_) during the reductive carboxylation of crotonyl-CoA with NADPH [29], which counter-balances the production of reducing equivalents during later steps in this pathway (resulting in a neutral net balance of reducing equivalents).

This result also confirms the observation that a RubisCO knockout mutant strain of *Rba. Sphaeroides *can grow photoheterotrophically on acetate which is in contrast to a similar *Rps. palustris *mutant where the utilization of the glyoxlate shunt requires the Calvin cycle as redox sink [33]. In fact, the computed minimum RubisCO flux for growth on acetate with the glyoxylate cycle (scenario S3 in Table 2) is very close to an experimentally determined RubisCO flux in *Rps. palustris *[10]. Interestingly however, both mutants were not capable to grow photoheterotrophically when malate was employed as sole carbon source. As discussed in the previous section, the Calvin cycle is needed as a redox sink when growing on malate in order to capture excess reducing equivalents liberated when precursor metabolites (e.g. PEP, pyruvate, acetyl-CoA, α-ketoglutarate) are synthesized. The excess of reducing equivalents arises from the required operation of enzymes such as the malate dehydrogenase or malic enzyme generating NAD(P)H. However, given the neutral redox balance of the EMCoA pathway when assimilating acetate to malate (see net stoichiometry above) it seems counterintuitive why growth on malate requires the Calvin cycle whereas growth on acetate does not. Our model shows that the rationale for this observation is related to an additional aspect of acetate metabolism. First of all, the application of the present PNSB model allowed us to easily simulate a metabolic network where the EMCoA pathway is actually operating in a RubisCO-deleted strain when growing on malate. In fact, the presence of the EMCoA pathway under this condition turned out to be not sufficient for releasing the mutant strain from deficient redox balancing and to restore growth. Instead, our analysis shows that the different growth capabilities of the *Rba. sphaeroides *RubisCO mutant on acetate vs. malate are exclusively related to the generation of just two precursors which are acetyl-CoA and α-ketoglutarate. Synthesis of one molecule of acetyl-CoA from malate (via pyruvate) is inevitably coupled to the formation of two NAD(P)H and in case of α-ketoglutarate (produced from two molecules of malate) even four reducing equivalents are generated. In contrast, when growing on acetate, acetyl-CoA can directly be produced from acetate without side production of reducing equivalents and α-ketoglutarate (produced from 2.5 molecules of acetyl-CoA) is coupled to formation of only two NAD(P)H. This means that growth of the RubisCO mutant is possible with acetate because of the lower burden of excess reductant when assimilating this substrate. In contrast, with malate, the higher amounts of reduction equivalents produced cannot be fixed by the RubisCO mutant irrespective of the (theoretical) presence or absence of the EMCoA pathway.

To summarize, the ability of a RubisCO mutant of *Rba. sphaeroides *to grow on acetate can be attributed to two network properties: (i) the EMCoA pathway has a neutral redox balance (in contrast to glyoxylate and CM cycle which produce reducing equivalents in their net balance). (ii) Synthesis of the two required precursors acetyl-CoA and α-ketoglutarate from acetate deliberates much less reducing equivalents than from other substrates such as malate.

We also noticed that, in principle, growth on malate would be possible in a RubisCO mutant of *Rba. spharoides *if we allowed the cells to produce acetyl-CoA via the pyruvate-formate-lyase (coupled to the excretion of formate excluded in scenario S2). This would make the Calvin cycle dispensable for growth on malate (but not with succinate as substrate), albeit this leads to loss of carbon and thus to a reduced biomass yield. Regulatory constraints seem to prevent the pyruvate-formate-lyase to function under photoheterotrophic conditions, since no formate production was reported experimentally [6]. Again, another mechanism to abolish essentiality of the Calvin cycle is to release hydrogen as discussed in the following section.

Furthermore, as mentioned above, even for the EMCoA pathway we could not determine a unique RubisCO flux and discussed instead the *minimal *possible flux (which is zero) whereas the maximal rate is non-zero. The reason is that there is a hypothetical futile cycle: if the two malate molecules produced via the EMCoA pathway would be reconverted to acetyl-CoA, the net balance would be the oxidation of one acetyl-CoA molecule to two molecules of CO_2 _and the formation of two reducing equivalents. Hence, if this cycle would run to a larger extent, redox balancing via the Calvin cycle (refixing lost CO_2 _and consuming excess reductants) becomes essential again. It is clear that an elevated combined operation of the EMCoA pathway and the Calvin cycle would represent a futile cycle consuming ATP and the cell needs a tight regulation, especially of the pyruvate dehydrogenase, to prevent such energy-wasting modes. Basically, such futile cycles could also arise in combination with the CM cycle and the glyoxylate shunt where a net oxidation of acetyl-CoA would demand an even higher flux through redox balancing pathways.

### Sensitivity of Calvin cycle flux with respect to other pathways

We now return to scenario S2 where we considered photoheterotrophic growth with some assumptions on inactive pathways whose reaction rates were set to zero. In this section we want to investigate the sensitivity of the RubisCO flux with respect to these fluxes indicating whether and how the Calvin cycle can be relieved or is even further burdened by the operation of these pathways. For this purpose, we take (as an example) scenario S2 (photoheterotrophic growth on succinate) and increase separately the flux of one of the previously fixed key reactions of certain pathways (see Table 1) from 0 to 0.1 and observe the change in the RubisCO flux as computed by FVA. Dividing this change by 0.1 we obtain the (at least locally valid) sensitivity of the RubisCO flux against the operation of the respective pathway.

We start with the reductive TCA and increase the flux of the α-ketoglutarate synthase from 0 to 0.1. As a consequence, the flux through the Calvin cycle decreases by 0.3 yielding thus a sensitivity of -3 (Table 3). As the operation of the reductive TCA consumes NADH and fixes CO_2 _it is clear that the reductive TCA can take over the role of the Calvin cycle - the high sensitivity indicates that this could be achieved in a very efficient way. *Rba. sphaeroides *does not possess the key enzymes of reductive TCA and although the significance of the reductive TCA in *Rs. rubrum *has been questioned [27] a very small activity in the latter could, in principle, explain why a RubisCO-lacking mutant of *Rs. rubrum *can grow on malate whereas a RubisCO-lacking mutant of *Rba. sphaeroides *cannot unless nitrogenase activity (dispensing excess reducing equivalents via hydrogen; see below) is elevated [5,9-11,27,39]. Table 2 shows that for growth on malate a RubisCO flux of 0.2 mmol per mmol substrate taken up would be required. A small reductive TCA flux of 0.067 mmol per mmol substrate uptake would thus enable the cell to endure a loss of RubisCO activity and to maintain redox balance with the help of this alternative pathway. However, it could also be that RubisCO mutants of *Rs. rubrum *use other or additional mechanisms to dispense reducing equivalents enabling them to grow on malate (cf. [27]).

**Table 3:** Sensitivity of the RubisCO flux with respect to the activity of other pathways under photoheterotrophic growth.

Pathways	Sensitivity of RubisCO flux
Reductive TCA (only in *Rs. rubrum*)	-3
Oxidative TCA	+3
Hydrogen release via nitrogenase	-0.53
Oxidative pentose phosphate pathway (only in *Rps. palustris*)	+1
Entner-Doudoroff Pathway (only in *Rba. sphaeroides*)	0

The sensitivities are computed with respect to scenario S2.

It is not surprising that the sensitivity of the RubisCO flux with respect to the oxidative TCA (key enzyme α-ketoglutarate dehydrogenase) has the same absolute value as the reversely operating reductive TCA but now with positive sign (+3; Table 3). The high sensitivity indicates the considerable higher load of the Calvin cycle if the TCA is running in the oxidative direction: it counteracts the loss of CO_2 _and the production of reducing equivalents by the oxidative TCA. This futile cycle composed of oxidative TCA and Calvin cycle must be avoided by the cell and in fact, as in many other bacteria, the activity of the α-ketoglutarate dehydrogenase is low under anaerobic conditions [41].

Another possible mechanism employed by PNSB for dispensing excess reducing equivalents under redox stress conditions (e.g. in RubisCO-lacking mutants) is uncontrolled expression of the nitrogenase complex (e.g. by adaptive evolution) which operates with an inherent hydrogenase activity [10,17]. Assuming an exclusive hydrogenase activity of the nitrogenase (without fixation of dinitrogen) we compute a relatively low sensitivity of -0.53 mmol RubisCO flux/mmol H_2 _released when growing on succinate. Importantly, the loss of electrons (not permitted in the original scenario S2) now also affects the CO_2 _net release (increases) as well as the biomass yield (decreases): we obtain the sensitivities -0.01 gDW biomass per mmol hydrogen released and +0.47 mmol CO_2 _net release/mmol hydrogen released, which are valid for all substrates. With these values we can almost perfectly reproduce CO_2 _and biomass yields experimentally determined for a nitrogenase-overproducing *Rps. palustris *strain growing on acetate and producing hydrogen [10] (data not shown).

It is noteworthy that, if the environmental conditions allow for a net fixation of dinitrogen, the loss of electrons is even larger since electrons are then not only released as hydrogen but are also consumed for the reduction of nitrogen [12,17].

Finally we want to study the sensitivity of the Calvin cycle flux with respect to the operation of the oxidative pentose phosphate pathway (oxPPP) in *Rps. palustris *and of the Entner-Doudoroff pathway (EDP) in *Rba. sphaeroides *on the flux through the Calvin cycle. As mentioned above and assumed in scenario S2, these pathways are inactive in *Rs. rubrum *due to the lack of a glucose-6-phosphate dehydrogenase. EDP and oxPPP are generally highly relevant for regenerating NADPH. They share the first two steps by which NADP is reduced to NADPH and 6-phoshphogluconate is produced as an intermediate. From here the two routes branch. In the oxPPP, this metabolite is oxidized to ribulose-5-phosphate entering the pentose phosphate metabolism (this reaction is coupled to the release of one CO_2 _molecule and to the reduction of NADP to NADPH). As an alternative, the EDP converts this 6-carbon intermediate to two 3-carbon metabolites pyruvate and glyeraldehyde-3-phosphate without further oxidation.

Computing the sensitivities of the RubisCO flux we observe that the sensitivities are indeed different for both pathways. For the oxPPP we compute a sensitivity of 1 (Table 3). Hence, if the cell increases the flux through the oxPPP for generating NADPH (with CO_2 _as side product) the RubisCO flux must increase with the same amount. In this way, the released CO_2 _is refixed and, at a later step in the Calvin cycle, 2 NADH are consumed leading to net electron balance of zero. Again, redox homeostasis forces this behavior. The combined operation of the oxPPP and the Calvin cycle could, in principle, be used as a virtual transhydrogenase whose net balance can be calculated as 2 NADH + 2 NADP + 3 ATP → 2 NAD + 2 NADPH + 3 ADP + 3 Pi. Whether this transhydrogenase-like operation is really employed by PNSB (when growing on organic acids) can be questioned due to the energetically wasteful consumption of 3 ATP (compared to the transhydrogenase). Moreover, many reactions and thus a large overall flux would be required to drive this cycle. In fact the activity of the oxPPP under photoheterotrophic conditions seems to be low [10].

In contrast, the sensitivity of the RubisCO flux with respect to EDP is zero (Table 3). Taking a closer look we can see that the EDP in combination with some gluconeogenic reactions can indeed also serve as virtual transhydrogenase. Starting and ending in glucose-6-phosphate it results in a net conversion of NADH + NADP + 1 ATP → NAD + NADPH + ADP + Pi. This cycle would not require refixation of CO_2 _via an increased RubisCO flux. Again, whether this cycle has any physiological relevance in *Rba. sphaeroides *(or in other representatives of PNSB), remains to be shown experimentally.

### Role of the transhydrogenase in *Rs. rubrum*

As *Rs. rubrum *does not possess the glucose-6-phosphate dehydrogenase it must use other mechanisms for producing NADPH (essentially required for biomass synthesis). The NADPH-producing malic enzyme shows low activity in *Rs. rubrum *when growing on succinate or malate [41]. Another potential supplier of NADPH, the isocitrate dehydrogenase reaction, has probably low fluxes (oxidative TCA is likely to be down-regulated under photoheterotrophic conditions as discussed above). Thus, the transhydrogenase remains the only catabolic reaction that can generate NADPH. The important role of the transhydrogenase for photoheterotrophic growth of *Rs. rubrum *was also discussed and experimentally supported by others [5,41]. Our model confirms this observation as a relatively high transhydrogenase flux (0.76 mmol (in direction of NADPH synthesis) per mmol substrate taken up) is computed in scenario S2 when fixing the rate of malic enzyme additionally to zero.

### Anaerobic growth in darkness (scenario S6)

For simulating fermentative growth in the dark we fix the rates of oxygen uptake and photosynthesis to zero while "opening" the gates for excretion of the products acetate, propionate, succinate and formate (scenario S6, Table 1). Initially we concentrate on fructose as a well-known substrate that can be fermented under anaerobic conditions. As for S1, an underdetermined network results. However, this time, no additional fluxes (besides a few trivial zero fluxes) can be determined uniquely. The reason is that ATP formation as well as NAD regeneration can be accomplished by different fermentative routes with different yields. As usual for fermentative metabolism the excreted fermentation products serve as flexible electron sinks. Interestingly, analysis of the feasible flux space for the scenario S6 with respect to biomass yield and CO_2 _excretion also revealed that there are flux distributions that allow a net-fixation of CO_2 _e.g. by employment of the Calvin cycle. However, fixation of CO_2 _does not lead to higher biomass yield but implies instead a higher yield of fermentation products (succinate, formate, acetate or propionate). Actually, the optimal biomass yield is only achievable without participation of ATP-consuming CO_2_-fixing pathways. Nevertheless, CO_2 _fixation may serve as a possible (but not essential) redox-balancing mechanism. How far these pathways are active at all under anaerobic conditions in the dark has to be investigated.

We also tested the capabilities of PNSB to grow on the organic acids malate, succinate and acetate anaerobically in the dark. As expected, fermentative growth on acetate is not possible, irrespective of the applied acetate assimilation pathway. Malate can be utilized (with very low biomass yields) under these conditions by employing the fumarate reductase as a mechanism for ATP generation. As a somewhat surprising result we found that, in principle, fermentative growth with succinate as substrate is also possible albeit with very low biomass yields. An essential requirement would be the release of hydrogen or the operation of either the Calvin cycle or (in *Rs. rubrum*) of the reductive TCA; these pathways would again serve as redox sinks. Net ATP synthesis would be facilitated by directing a large proportion of the succinate taken up to propionate. Assuming that enzyme propionyl-CoA-carboxylase can operate as a reversible enzyme this allows the conversion of succinate to propionate via succinyl-CoA and methylmalonyl-CoA with a net synthesis of one ATP per molecule succinate. Of course, if the reaction catalyzed by propionyl-CoA-carboxylase is irreversible *in vivo *or if the decarboxylation of methylmalonyl-CoA is mediated (by another yet non-annotated enzyme) without generation of ATP (like the methylmalonyl-CoA-decarboxylase present in *E. coli*) succinate can not be utilized anaerobically in the dark. It remains thus a speculation whether succinate can be fermented (with very low growth rates) or not, however, the model provides an unbiased tool to detect such unexpected behaviors.

### Aerobic growth in darkness (scenario S7)

Scenario S7 specifies a general scenario for aerobic growth. Photosynthesis, Calvin cycle, fumarate reductase, reductive TCA and nitrogenase reaction were disabled due to known regulatory constraints and we assume that no products other than CO_2 _are excreted. This scenario is again underdetermined but - due to the larger number of fixed fluxes - with less degrees of freedom compared to photoheterotrophic growth (S1). Nevertheless, in contrast to S1, up to some trivial zero fluxes, no additional rates can be determined uniquely by FVA in these aerobic scenarios for any of the substrates. The reason is that O_2 _as electron acceptor eliminates the strict coupling of carbon and redox balancing. Oxygen serves virtually as an unlimited redox sink (thereby generating energy in form of ATP) and there is no need to install other pathways to regenerate NAD even if a substrate is more reduced than required for biomass and ATP synthesis. As the total ATP demand is unknown (the maintenance demand (i.e. the rate of the pseudo reaction *ATPdrain*) is not specified) we cannot predict how much of the substrate is directed to the oxidative TCA generating NADH for driving respiration. Even if we would know (or estimate) the maintenance demand this would not lead to a unique determination of unknown flux values as there are futile routes and pathways with different ATP yields (e.g. ubiquinol oxidase vs. cytochrome oxidase).

### Capabilities of PNSB for biotechnological hydrogen production

PNSB have been thoroughly studied for their capacity to produce H_2 _from a variety of organic compounds at high yields (reviewed e.g. [43]). We therefore chose biohydrogen to demonstrate the value of our stoichiometric model for biotechnological applications by (a) testing the theoretical capabilities of PNSB for biohydrogen production and by (b) identifying genetic intervention strategies that could further increase the H_2 _yield. Generally, there are four known mechanisms of H_2 _release that can be used by PNSB [44,45]: nitrogenase (see previous sections), uptake hydrogenase (in H_2 _production direction), formate hydrogenlyase and a CO-linked hydrogenase (the latter is not considered herein). Wild types of the three considered PNSB species do not produce H_2 _in significant amounts at high-light photoheterotrophic conditions (otherwise, experimentally determined biomass yields would be significantly smaller than the predicted biomass yields - which is not the case; cf. Table 2). The H_2 _production rate depends on three major factors: (i) electron availability (required by all four mechanisms); (ii) demand and availability of ATP (e.g. relevant for H_2 _production via nitrogenase); and (iii) regulation (or deregulation) of the expression of enzyme complexes involved in H_2 _synthesis (e.g. uncontrolled expression of nitrogenase; see above).

To increase the availability of electrons it is straightforward to knockout electron competing pathways such as the Calvin cycle. To evaluate the effects of this knockout, we applied the constraints as described with scenario S2 but fixed the RubisCO flux to zero while allowing H_2 _release via nitrogenase or hydrogenase. We did that for all substrates (for acetate we enabled the glyoxylate cycle for *Rps. palustris *and the EMCoA pathway for *Rs. rubrum *and *Rba. sphaeroides*) and applied FVA to evaluate the range of possible H_2 _yields (Table 4). For reasons discussed in the previous sections, the knockout of RubisCO results in obligatory coupling of hydrogen release and biomass production for all substrates except for acetate assimilation via the EMCoA pathway where reducing equivalents are not necessarily available in excess. For the other substrates, the H_2 _release is proportional to the RubisCO flux in the wild type (Table 2). Since this electron sink is no longer available, H_2 _production becomes the only possibility to balance the reducing equivalents. However, the H_2 _yield is far away from being optimal in this scenario. A possibility to further improve electron availability would be the activation of an oxidative pathway, e.g. the oxidative pentose phosphate pathway in *Rps. palustris *or the oxidative TCA (available in all three species). These pathways are normally repressed under photoheterotrophic conditions (for reasons discussed in previous sections) but a targeted deregulation of these pathways could, in principle, lead to the maximal possible H_2 _yields (Table 4). (Note that the maximally achievable H_2 _yields can also be calculated from the reduction degree of the carbon atoms in the substrate relatively to CO_2_. For example, the latter is 3.5 for each C atom in succinate implying that for a complete oxidation 14 electrons and thus a maximal yield of 7 mole H_2 _per mole succinate could be generated).

**Table 4:** Theoretical ranges of hydrogen yields for photoheterotrophic growth on different substrates and with different genetic modifications.

Substrate	ΔRubisco	ΔRubisco, +oxPPP/+oxTCA	**max Y**_**H2**_
Succinate	1.38	1.38 - 7	7 (1.75)
Malate	0.38	0.38 - 6	6 (1.5)
Propionate	1.38	1.38 - 7	7 (2.33)
Fructose	0.75	0.75 - 12	12 (2)
Acetate (Glyox)	0.51 - 4	0.51 - 4	4 (2)
Acetate (EMCoA)	0-4	0-4	4 (2)

ΔRubisco: RubisCO knockout, +oxPPP/+oxTCA: activated oxidative pentose phosphate pathway/activated oxidative TCA. Yields are given as mole hydrogen per mole substrate, yields in parentheses (last column) are given as mole hydrogen per mole carbon.

Note that the maximal yield determined by FVA is not identical to the value calculated by simply balancing H atoms derived from the substrate. For example, the theoretical H_2 _production yield with fructose is generally given as 6 mol H_2 _per mol substrate (corresponding to 12 H atoms present in fructose). However, as listed in Table 4, the theoretical maximum yield is in fact 12 mol H_2_/mol substrate. This much higher yield becomes possible because electrons derived from oxidation of the substrate C atoms to CO_2 _combine with protons that originate not from the substrate itself but e.g. from H_2_O in hydroxylation reactions.

The release of H_2 _could be mediated either by the hydrogenase activity of the nitrogenase (at the expense of 4 mole ATP per mole H_2 _released) or by the reverse direction of the uptake hydrogenase (without consumption of ATP). Since the main excretion route of H_2 _is via the nitrogenase, photoheterotrophic conditions have the advantage that the required ATP is generated by photosynthesis. Thus, no carbon needs to be metabolized for ATP generation and higher yields compared to fermentative conditions can be achieved. However, H_2 _excretion could also be driven (at least partially) by the uptake hydrogenase functioning in reverse direction or by an alternative ATP independent system, e. g. the formate hydrogenlyase. Whereas the activity of the uptake hydrogenase in reverse direction is questionable, all genes and a significant activity of the formate hydrogenlyase enzyme complex have been shown to be present in *Rs. rubrum *and *Rps. palustris *[46,47]. H_2 _production would then also be possible by fermentation in the dark, similar to *E. coli *producing H_2 _via formate hydrogenlyase [48]. However, this operative mode would also require appropriate strain design since the excretion of competing fermentative products will lower the H_2 _yield in practice.

We emphasize here once more that FVA allows one to determine the theoretically achievable H_2_. Whether these fluxes can be realized in nature is of course dependent on additional kinetic and thermodynamic constraints. For example the complete conversion of organic compounds to H_2 _and CO_2 _by fermentative metabolism is thermodynamically unfavourable. In this respect PNSB appear to be more promising since they can use light energy for activating the release of H_2 _and, in contrast to other photosynthetic microorganisms, they do not produce oxygen which severely inhibits hydrogenase and nitrogenase enzymes

## Conclusions

We presented a stoichiometric model of the central metabolism of PNSB and analyzed its properties with focus on two key issues related to PNSB metabolism: (i) stoichiometric constraints related to redox balancing under photoheterotrophic conditions and (ii) the role of the proposed acetate assimilation pathways. Balancing of reducing equivalents and carbon fluxes imposes tight constraints on feasible flux distributions under phototrophic conditions. As one consequence a unique and specific biomass yield and CO_2 _release can be computed for a given substrate despite a large degree of freedom with respect to utilizable catabolic pathways. Biologically, this means that, in order to balance reducing equivalents and carbon metabolites simultaneously, the cell has to comply with substrate-specific ratios of CO_2 _release and biomass yield per substrate taken up. These results confirm experimental data and the well-known observation that the CO_2 _production rate correlates with the oxidation state of the chosen substrate. Another important aspect we studied is the importance of the Calvin cycle when PNSB grow photoheterotrophically on carbon substrates. Our model reproduces and explains various, partially species-specific experimental results showing that the Calvin cycle serves as an essential redox sink for many substrates even if carbon dioxide is released in the net balance. We could also discuss quantitatively the role of other pathways which could either function as alternative redox sinks or which liberate further reducing equivalents implying an even larger flux through the Calvin cycle.

We also investigated putative acetate assimilation routes in PNSB. As one key result, our model suggests that *Rba. sphaeroides *uses the EMCoA pathway (and not the citramalate cycle) under photoheterotrophic conditions because only this pathway can explain the fact that a Rubisco mutant can grow on acetate. However, the virtual incorporation of an operating EMCoA pathway in a scenario of phototrophic growth with malate demonstrated that the activation of this pathway could not compensate for RubisCO in a mutant strain if malate is the substrate and that instead metabolic constraints related to precursor synthesis can explain this distinct behavior.

In summary, metabolic network modeling enabled us to interpret accumulated biological knowledge and to get a deeper understanding of global redox balancing mechanisms in PNSB. The model provides a rationale for explaining various experimental findings and several new testable hypotheses could be formulated. Furthermore, as PNSB are promising candidates for biotechnical applications including the production of biohydrogen, biopolymers, or porphyrins [4,10,49-53], our model could become a valuable tool for the *in silico *design of genetically-engineered bacterial strains.

## Methods

### Reconstruction of the stoichiometric model of the central metabolism in PNSB

The reconstructed metabolic network model is a "master model" comprising the major catabolic pathways of *Rs. rubrum*, *Rba. sphaeroides *and *Rps. palustris*. We used available information from biochemical literature, annotated genomes [4,54] and the KEGG database to reconstruct the network. As explained in the main text, the focus of this work was on aspects of the central carbon metabolism and to understand the distribution of major metabolic fluxes under different environmental conditions. We therefore included catabolic and amphibolic pathways as complete as possible. Biosynthetic routes for the production of building blocks and macromolecules were modeled in a simplified manner by using lumped reactions. This representation is still fully sufficient to compute quantitatively precursor effluxes into biomass synthesis.

The starting point of our reconstruction was the stoichiometric model presented in [20]. As this model was originally developed as a minimal model to discuss theoretical issues of metabolic flux analysis (and less biological aspects), it had to be revised and extended considerably. For instance, the number of reactions increased from 76 to 142.

We screened the KEGG database for specific information on the availability of reactions of central metabolic pathways in the three considered species and included them in the model. The few reactions that were only available in one or two of the three species were marked accordingly (see Figure 1; each species possess two reactions not contained in the other two representatives). Subsequently, we screened PNSB-related literature to identify and include relevant metabolic reactions and pathways that were not contained in the KEGG database but where evidence for their existence was given in the literature. This pertains in particular to the recently identified acetate assimilation pathways. The model was further expanded with biosynthesis routes for amino acids and other building blocks (nucleotides, fatty acids, etc.). These anabolic pathways were modeled in a condensed manner (reactions in a linear chain are lumped to an overall reaction). Finally, we integrated a biomass synthesis (pseudo) reaction forming biomass from ATP, NADPH, precursors and building blocks from the central metabolism. Additionally, reactions for uptake and excretion of typical substrates and products, respectively, were incorporated.

The model consists of 143 reactions and 119 balanced (internal) metabolites. Figure 1 shows the catabolic and amphibolic pathways of the central metabolism (anabolic pathways are not displayed). The complete network definition together with the biomass composition is given in the Additional File 1. The model is also available in SBML format in the Additional File 2.

In the following we discuss some specific biological details of the reconstructed metabolic network.

#### Substrates and products

As described above, PNSB are very versatile and can utilize a wide range of different substrates. Here we concentrated on succinate, malate, propionate, acetate and fructose as the main carbon sources for heterotrophic growth. The spectrum of utilizable sugars differs among the three chosen species. For example, *Rs. rubrum *lacks a glucose transporter and can therefore not grow on glucose. We used fructose as representative for hexose substrate. Its uptake is mediated by a fructose-specific PTS-system [55]. As possible products, we accounted for excretion of succinate, acetate, formate and propionate. Since the exchange of CO_2 _with the environment is a passive diffusion process, a reversible exchange reaction for CO_2 _was incorporated. For autotrophic growth, we considered hydrogen (H_2_) and carbon dioxide (CO_2_) as source of electrons and carbons, respectively. Uptake of sulfate (SO_4_), ammonia and inorganic phosphate is also considered in the model.

#### Central catabolic pathways

The proposed stoichiometric model accounts for the reactions of glycolysis, gluconeogenesis, pentose phosphate pathway (the complete oxidative branch is only present in *Rps. palustris*), tricarboxylic acid cycle (TCA) and the Entner-Doudoroff pathway (only in *Rba. sphaeroides*). PNSB can fix carbon dioxide via the Calvin cycle whose specific reactions, including the carbon fixing step catalyzed by the ribulose-1,5,-bisohosphate carboxylase/oxygenase (RubisCO), were also incorporated. *Rs. rubrum *has been shown to have enzymatic activities of the reductive TCA, notably the ferredoxin-dependent reactions of the α-ketoglutarate synthase and pyruvate synthase [26]. On the other hand, *Rs. rubrum *does not possess a glucose-6-phosphate dehydrogenase and therefore lacks both the oxidative pentose phosphate pathway and the Entner-Doudoroff pathway. As anaplerotic reactions pyruvate carboxylase, malic enzyme, PEP carboxykinase and the glyoxylate shunt (only in *Rps. palustris*) were included in the network. In contrast to *Rps. palustris *the lack of isocitrate lyase in *Rs. rubrum *and *Rba. sphaeroides *prevents these two species to use the conventional glyoxylate shunt for anaplerotic replenishment of TCA cycle precursors during growing on acetate. For *Rba. sphaeroides*, Erb and co-workers have recently elucidated an entirely novel mechanism for acetate assimilation - designated as the ethylmalonyl-CoA (EMCoA) pathway, according to the characteristic intermediate of the reaction sequence [29]. Based on genome sequence information, all necessary enzymes of the EMCoA pathway were found to be also present in *Rs. rubrum *[30]. For *Rs. rubrum *however, an alternative citramalate (CM) cycle has been proposed by Ivanovsky et al. [32] as a fundamentally different pathway for acetate assimilation. Moreover, enzyme activities of the citramalate cycle have been demonstrated in *Rba. sphaeroides *[31]. All three variants for acetate assimilation were incorporated into the metabolic network model for *in silico *analysis of their consequences for global metabolic properties.

The model also contains a reaction mimicking consumption of ATP for maintenance processes.

#### Photosynthesis and electron transport chain (ETC)

ATP is mainly generated by electron transport phosphorylation. Under aerobic conditions, the transfer of electrons from NADH to the final electron acceptor oxygen (either via an ubiquinol- or an cytochrome c_2 _oxidase) is coupled to pumping of protons generating a proton motive force that can be used to drive the ATP synthase. When exposed to light under anaerobic conditions, cyclic photophosphorylation becomes the operational mode of the ETC for ATP synthesis. Thereby, electrons are cycled from ubiquinol to cytochrome c_2 _(via bc_1 _generating proton-motive force) and back to ubiquinone (promoted by the reaction center at the expense of absorbed photons). A detailed model-based analysis of the different operational modes of the ETC in PNSB was given elsewhere [42]. All reactions and stoichiometries of this detailed model were incorporated in the model presented herein.

Due to their important role in redox balancing, we also included the reactions of the membrane-bound transhydrogenase and nitrogenase. The latter converts N_2 _under consumption of ATP and NADH to NH_4 _and its activity is inherently coupled to the release of hydrogen. We also included a reaction mimicking the release of hydrogen via the nitrogenase without a net fixation of dinitrogen [10].

#### Biomass formation

Some data on the biomass composition and estimated precursor demands for synthesizing biomass components in PNSB are available in the literature [10,20]. For our model we took the macromolecular biomass composition published in [20] (see Table 3 in Additional File 1). The following biomass components were included: proteins (50%), lipids (16%), RNA (16%), PHB (5%), DNA (3%), lipopolysaccharides (3%), glycogen (2%), peptidoglycan (2%), polyphosphates (2%) and bacteriochlorophyll (1%). The unavoidable uncertainty in the biomass composition (which may also change for different conditions) results in approximated values for the effluxes of precursors into biomass synthesis. Again, this variability has no impact on the qualitative results presented in this paper.

#### Reaction reversibilities

Uptake and excretion reactions were set to irreversible (except for carbon dioxide). Several reactions (e.g. flux through RubisCO reaction in the Calvin cycle) are considered to be irreversible under physiological conditions and their reaction rates are thus constrained to be non-negative.

### Flux variability analysis (FVA)

A central assumption of metabolic network analysis is that the system is in steady state, i.e. the concentrations **c **of internal metabolites are constant [21]. With the *m *× *q *stoichiometric matrix **N **(*m *rows: metabolites; *q *columns: reactions with the respective stoichiometric coefficients for the participating metabolites) and the vector **r **of reaction rates (fluxes), this can mathematically be formulated as a system of linear (metabolite balancing) equations

(1) $$\frac{dc}{dt}=0=N\;\cdot r$$

The reaction rates are typically expressed in mmol per gram dry weight and hour [mmol/(gDW⋅ h)]. Under physiological conditions, each reaction will have a limited maximal flux due to thermodynamic constraints. The exact *in vivo *limits are usually not known, except that irreversible reactions cannot have a negative flux. Nevertheless, it makes sense to restrict the reaction rates to a high value (e.g. 100 mmol/(gDW· h)) resulting in the additional constraints

(2) $$\begin{array}{lll} 0 & \leq r_{j}\leq 100\;\text{if reaction }j\text{ is irreversible}\; & \text{(1)}\\ -100 & \leq r_{j}\leq 100\;\text{if reaction }j\text{ is reversible}\; & \text{(2)}\\ & \; & \text{(3)} \end{array}$$

Equation (1) and inequalites (2) usually define an underdetermined system and, hence, no flux can be calculated uniquely. However, additional constraints arise when some (*k*) reaction rates $\left\{r_{m_{1}},\;...,r_{m_{k}}\right\}$ with selected reaction indices *m*_*1*_, ..., *m*_*k *_∈ {1,..., *q*} are known, e.g. because their flux values $\left\{v_{m_{1}},\;...,v_{m_{k}}\right\}$ have been measured experimentally or because they can be set to zero as they cannot operate under a given environmental condition:

(3) $$\begin{array}{c} r_{m_{1}}=v_{m_{1}}\\ \vdots \\ r_{m_{k}}=v_{m_{k}} \end{array}.$$

Some of the unknown rates might then become calculable. For calculating uniquely determined reaction rates or for identifying feasible ranges of the fluxes we will use *Flux Variability Analysis *(FVA, [56]), an approach that is based on linear programming. Separately for each unknown reaction *r*_*i *_we search for the minimal possible flux and for the maximal possible flux that a reaction can carry in any flux distribution that is consistent with the constraints (1)-(3). The corresponding optimization problem reads

(4) $$\begin{array}{c} r_{i,\min}=\underset{r}{\min}r_{i}\;\;\;\;\;\;\;\;\;or\;\;\;\;\;\;\;r_{i,\max}=\underset{r}{\max}r_{i}\\ s.t.\\ 0=N\cdot r\\ r_{m_{1}}=v_{m_{1}}\\ \vdots \\ r_{m_{k}}=v_{m_{k}}\\ 0\leq r_{j}\leq 100\;\;if\;reaction\;j\;irrevesible\\ -100\leq r_{j}\leq 100\;\;if\;reaction\;j\;revesible \end{array}$$

The information we get from the 2·*u *optimizations (*u *= number of unknown fluxes) is the physiologically feasible flux range for the unknown reactions. Moreover, if the computed minimal and maximal rate of a reaction coincide, *r*_*i,min *_= *r*_*i,max*_, the reaction rate follows to be uniquely determined

Note that FVA as described above does not make any assumption about biological objectives, in contrast to flux balance analysis [24]. The objective function in (4) only serves as a tool to identify the feasible flux ranges. However, the upper bound of the growth reaction identified by FVA is equivalent to the optimal molar growth yield (gDW per mmol substrate) achievable for the given constraint. This approach was introduced under the term flux-spectrum and used, for example, to estimate flux distributions in CHO cells [57].

All computations presented in this study were performed with our software *CellNetAnalyzer *[58], a MATLAB toolbox with graphical user interface facilitating, among other things, metabolic network analysis (see Figure 2). It can be downloaded from http://www.mpi-magdeburg.mpg.de/projects/cna/cna.html and the network project files will be made available on this site (within the CNA model repository). CNA has its own FVA functionality and uses the glpk solver for flux optimization.

## Authors' contributions

Conceived and planned the study: SK. Carried out the study: OH, SK. Interpretation of the results: OH, HG, SK. All authors read and approved the final manuscript.

## Supplementary Material

Additional file 1 **Network model of PNSB**. Full definition of the stoichiometric network model. Click here for file

Additional file 2 **Network model of PNSB in SBML format**. Network model of PNSB in SBML format. Click here for file
